# Robust gene expression and mutation analyses of RNA-sequencing of formalin-fixed diagnostic tumor samples

**DOI:** 10.1038/srep12335

**Published:** 2015-07-23

**Authors:** Stefan Graw, Richard Meier, Kay Minn, Clark Bloomer, Andrew K. Godwin, Brooke Fridley, Anda Vlad, Peter Beyerlein, Jeremy Chien

**Affiliations:** 1 Department of Cancer Biology, University of Kansas Medical Center, 3901 Rainbow Blvd, Kansas, 66160 Kansas City, USA; 2 Department of Laboratory Medicine and Pathology, University of Kansas Medical Center, 3901 Rainbow Blvd, Kansas, 66160 Kansas City, USA; 3 Genome Sequencing Facility, University of Kansas Medical Center, 3901 Rainbow Blvd, Kansas, 66160 Kansas City, USA; 4 Department of Biostatistics, University of Kansas Medical Center, 3901 Rainbow Blvd, Kansas, 66160 Kansas City, USA; 5 Department of Obstetrics, Gynecology & Reproductive Sciences, University of Pittsburgh, 4200 Fifth Ave, Pennsylvania, 15260 Pittsburgh, USA; 6 Department of Bioinformatics and Biosystems Technology, Technical University of Applied Sciences Wildau, Hochschulring 1, 15745 Wildau, Germany

## Abstract

Current genomic studies are limited by the availability of fresh tissue samples. Here, we show that Illumina RNA sequencing of formalin-fixed diagnostic tumor samples produces gene expression that is strongly correlated with matched frozen tumor samples (r > 0.89). In addition, sequence variations identified from FFPE RNA show 99.67% concordance with that from exome sequencing of matched frozen tumor samples. Because FFPE is a routine diagnostic sample preparation, the feasibility results reported here will facilitate the setup of large-scale research and clinical studies in medical genomics that are currently limited by the availability of fresh frozen samples.

Cancer is a generic term for a large group of malignant diseases that can affect any part of the body. It is characterized by invasive, abnormal growth which grows beyond the usual boundaries and spreads to adjoining or distant organs (metastasis). It is important to analyze and characterize genomic structural, sequence and expression variations that cause cancer or are associated with cancer in order to advance our knowledge of the disease and to develop new treatments for cancer^1^. The rapid development in high-throughput sequencing technologies is enabling the characterization of genome-wide alteration in cancer at single base resolution. It is now feasible to sequence entire cancer genomes from a large number of samples in a timely and cost-efficient manner^2^. Characterizing genomic changes in cancer is the key to the discovery of novel therapeutic targets. It is imperative to have a large number of samples from cancer patients to comprehensively and accurately characterize the genomic changes to distinguish true driver mutations from background passenger mutations^3^,^4^.

To identify somatic mutations associated with various cancer subtypes and clinical behavior, it is necessary to have the clinical information on the disease progression and patient outcome^5^. Large-scale efforts are underway to characterize somatic mutations across a number of different cancers so that comprehensive knowledge can be constructed to advance personalized medicine. However, at present these efforts rely on samples stored as fresh frozen (FF) tissues^6^,^7^,^8^,^9^,^10^,^11^. A major challenge in these studies is obtaining large numbers of fresh tissue samples that also have long-term follow up clinical information on disease progression and outcome^5^.

Formalin-Fixed Paraffin-Embedding (FFPE) has been the standard diagnostic sample preparation for pathologists for decades. This resource offers a potentially large volume of biospecimens to match disease and normal tissues for large-scale correlative studies^12^. Large FFPE archives represent an opportunity to sequence samples for retrospective studies to investigate the complex genetic changes underlying tumor progression, treatment response, therapy resistance and disease outcome. It is technically challenging to perform sequencing analyses on DNA isolated from FFPE samples because of damage induced by the fixation process^13^. RNA-sequencing from FFPE sample represents an even greater challenge because RNA is more labile than DNA.

Previous studies have successfully used DNA from FFPE samples for copy number analysis and mutation detection using targeted sequencing of single genes^14^,^15^, as well as the whole exome^16^,^17^,^18^,^19^ and whole genome^20^,^21^. However, limited studies are available for RNA sequencing from FFPE tissue samples because of the concern that RNA in FFPE samples may not be suitable for sequencing. In addition, these studies are limited by the lack of verification using orthogonal platforms, effect of transcript length and gene expression levels on RNA-sequencing results and analysis of sequence variations^22^.

In this study, we performed a comprehensive analysis of RNA-sequencing data from six pairs of FF/FFPE tumor samples. We used NanoString technology as an orthogonal verification platform to compare gene expression between FF and FFPE. Finally, we used SNPiR bioinformatics tool and customer filters to analyze sequence variations from RNA-sequencing and used DNA-sequencing data for verification purpose. These analyses indicate the feasibility of performing comprehensive genomic characterization of tumor samples from archived FFPE samples.

## Materials and Methods

### Materials

Six matched pairs of FF and FFPE ovarian tumor samples have been processed for RNA sequencing and DNA whole exome sequencing. RNA from these six pairs were also subjected to the NanoString gene expression analysis. Fresh tumor samples served as references to validate mutations and gene expression in FFPE samples (listed in Table S1). Additionally seven normal fallopian tube samples from GTEx have been used to identify differentially expressed genes in FF and FFPE tumor samples. These data sets will be available through dbGaP.

### Methods

#### Nucleic Acids Extraction

RNA and DNA from FFPE tumor samples were extracted using Qiagen FFPE All-Prep kit. Five micron slices of FFPE specimens were dewaxed using Deparaffinization Solution (Qiagen, Valencia, CA). DNA extractions were done using Qiagen All Prep DNA/RNA FFPE Kit (Qiagen, Valencia CA), according to Qiagen’s supplementary protocol for FFPE tissue (QIAamp DNA FFPE Tissue Handbook). We implemented the following protocol changes: All FFPE samples were digested with 20 μL Proteinase K shaking overnight at 56 °C and then supplemented with an additional 15 μL for an additional hour for complete protein digestion. Samples were eluted in 50 μL elution buffer. RNA and DNA from FF tumor samples were extracted using Trizol (Invitrogen) and Qiagen DNAeasy kit, respectively. RNA quality was determined by Agilent Bioanalyzer.

#### RNA library preparation

Stranded Total RNA library preparations were prepared using the Illumina TruSeq Stranded Total RNA LT kit (with Ribo-Zero Human/Mouse/Rat). 500 ng of FFPE RNA was used to initiate the ribosomal RNA reduction. To avoid over fragmentation of FFPE RNA, the standard 8 second fragmentation time for ribosomal reduced RNA was varied from 0 to 4 seconds to accommodate the average size distribution of each degraded FFPE RNA sample as determined from the Agilent Bioanlayzer quality control run. Fragmentation time was based on Agilent Bioanalyzer size range examples provided in the Alternate Fragmentation Protocols—Appendix A of the Illumina TruSeq Stranded Total RNA Sample Preparation Guide (part#15031048 Rev. D April 2013). The remaining stranded library preparation were performed according to the kit instructions. 500 ng of total RNA from FF samples was used with Illumina TruSeq Stranded mRNA Sample Preparation Kit. Validation of the library preparations was performed on an Agilent Bioanalyzer using the DNA1000 kit. Libraries were quantified using a Roche LightCycler96 with FastStart Essential DNA Green Master mix. Library concentrations were adjusted to 4 nM and pooled for multiplex sequencing. Pooled libraries were denatured and diluted to 15 pM then clonally clustered onto the sequencing flow cell using the Illumina cBOT Cluster Generation Station and TruSeq Paired-End Cluster Kit v3-cBot-HS. The clustered flow cell was sequenced on the Illumina HiSeq2500 Sequencing System using the TruSeq SBS Kit v3-HS.

#### DNA library preparation

All libraries were prepared from 50 ng of genomic DNA using the Nextera DNA Sample Prep Kit (Illumina, San Diego, CA) following the manufacturer’s protocol. Sample specific indexes were added during 10 cycles of PCR amplification. Excess primers and primer dimers were removed using Agencourt AMPureX beads (Beckman Coulter, Danvers, MA) at a 0.8X bead ratio. Libraries were quantified by qPCR using primers specific to Illumina adaptor sequences and library size was assessed on a high sensitivity chip run on the Agilent 2100 Bioanalyer (Agilent, Santa Clara, CA). All sample libraries underwent targeted enrichment for the whole exome per the Nextera Enrichment Sample Prep Protocol (Illumina, San Diego, CA). Illumina’s TruSeq Exome pool consisting of 90-mer biotinlyated oligos was used to enrich for the whole exome, covering known protein-coding genes, 5′ and 3′ UTRs, microRNA, and other non-coding RNA (62 Mbases total).

#### Bioinformatic analyses

**Figure 1:**
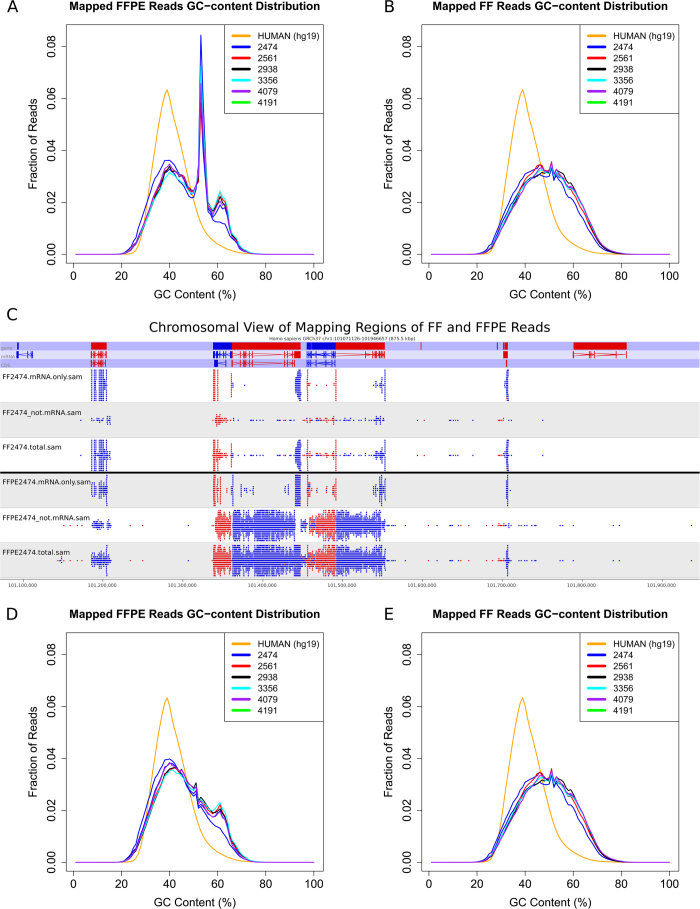
GC content distribution before and after applying filter on FF and FFPE samples. The GC-content distribution indicates abnormal GC peak (blue line) in FFPE (A) but not in FF (B) samples. The GC content of the human reference genome is plotted in red line. To understand why GC content in FFPE sample show an abnormal peak, mapped reads were visualized by SeqMonk. The representative chromosome view shows three rows of data tracks for each sample: (1) mRNA.only contains only reads in mRNA regions, (2) not.mRNA contains all reads that did not align to exons, (3) total contains all reads. Strand information is shown in red and blue. FFPE RNA sequencing produces reads that also map to intronic regions. When reads mapping to intronic regions in both FF and FFPE were filtered out, abnormal GC peak in FFPE samples were removed (D) without affecting the GC-content distribution of FF samples (E).

**Figure 2:**
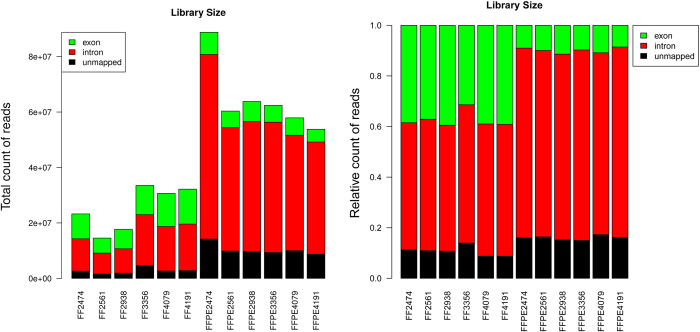
Fraction of reads mapping to various parts of the genome. Ribosomal RNA-depleted total RNA library preparation for FFPE RNA resulted in larger fraction of reads mapping to intronic regions (both absolute and relative fraction) compared to mRNA library preparation for FF RNA.

**Figure 3:**
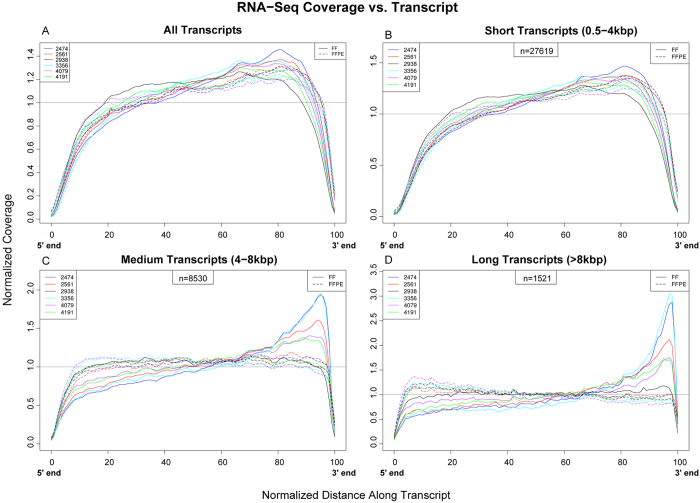
Coverage along the normalized transcript length. Coverage plots along the normalized transcript length for all transcripts (A) and for three different transcript length categories (B: 0.5–4 kbp, C: 4–8 kbp, D: >8 kbp) are shown to determine the effect of transcript length on the coverage along the entire transcript. The samples are color coded (e.g. FF2474&FFPE2474: blue), while the storage types are shown with different line styles (FF: solid; FFPE: dashed). The results show a discernable 3′ bias in FF samples while uniform coverage is observed in FFPE samples.

**Figure 4:**
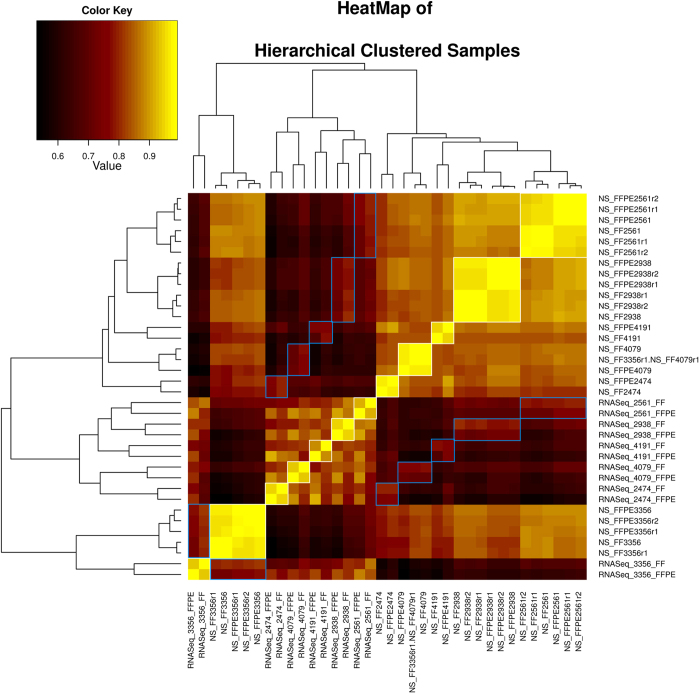
Clustered heat map to visualize correlation matrix. Heat map of clustered correlation matrix (RNA-seq&NanoString data sets from paired FF&FFPE samples) shows strong correlation in gene expression between paired FF and FFPE samples. Color key was adjusted to minimal and maximal values to differentiate the differences. Dendrogram illustrates the relationship-distance between samples. Associated samples (e.g. FF2474 and FFPE2474) from the data sets produced with the same technology (either RNA sequencing or NanoString) are highlighted with a white frame, whereas associated samples from different technologies are highlighted with a blue frame. Sample NS_FF3356r1.NS_FF4079r1 was a replicate sample that was mislabeled as FF3356r1 during NanoString analysis. However, clustering analysis correctly identified it as FF4079r1. Note that correctly labeled FF3356r1 clustered with other FF3356 and FFPE3356 replicates.

**Figure 5:**
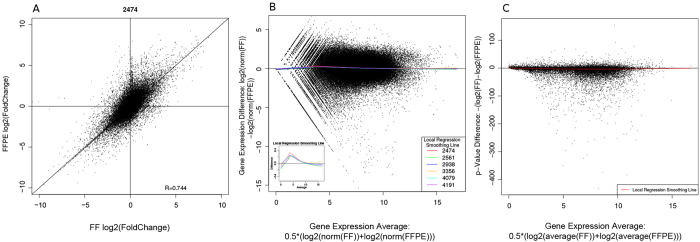
Differential gene expression correlation. Panel A shows a representative scatter plot of fold-change in gene expression in matched FFPE (y-axis) and FF (x-axis) tumor samples compared to seven normal samples. Each point represents a log2 (fold-change) value for a gene. R = 0.744 indicates strong correlation of fold-change in gene expression between matched FF and FFPE tumor samples compared to the gene expression in normal samples. Panel B presents a MA-plot, showing the normalized gene expression difference over the normalized average gene expression. A locally weighted smoothing regression was calculated for each sample and color coded. All six locally weighted smoothing regressions are also shown (zoomed in) as the inset. The results indicate consistently higher gene expression in FF samples compared to FFPE samples for genes with averaged log2 expression values of <10. Panel C shows a modified MA-plot. The differences in p-values derived from differential gene expression analysis is plotted against normalized average gene expression to investigate the bias in p-values for genes that are expressed at different levels. The results indicate no discernable effect of gene expression levels on the p-values from differential gene expression analysis of FF or FFPE samples compared to normal samples.

**Figure 6:**
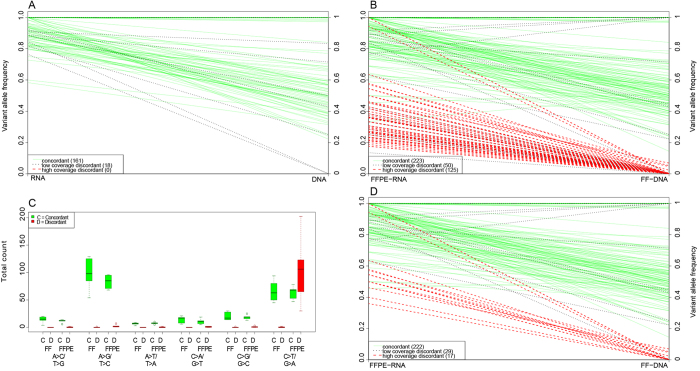
Variant allele frequencies and FFPE artifacts. Panel A, B and D show the comparison of variant allele fraction in FF or FFPE RNA (left y-axis) and FF DNA (right y-axis). Each line represents the relationship of one variant in comparison. The color code indicates whether the mutations is concordant (green), discordant (red), or ambiguous (black) because of low DNA coverage. Panel A shows the comparison between RNA with DNA from fresh frozen sample (2474). Most of the variants detected in RNA samples are concordant with variant calls from DNA. No discordant calls were observed. Eighteen variant calls from RNA sequence data were considered ambiguous because the same variants were not called in DNA due to low coverage. Panel B shows the comparison of variant allele fraction in RNA and DNA from paired FFPE and FF samples (2474), respectively. High degree of discordant calls were observed. Panel C shows the distribution of various substitutions in FF and FFPE samples. Substitutions that are concordant between paired FF and FFPE samples are indicated by green and discordant substitutions are indicated by red. The majority of discordant calls are observed as C>T or G>A substitutions, suggesting a documented FFPE artifact. Results from Panel D indicate that a small fraction of discordant calls remained after removing the C>T or G>A substitutions present at <0.5 fraction in FFPE RNA sequencing data sets. Note that the remaining discordant calls with allele fraction <0.5 are other types of substitutions.

To check the general quality of reads generated from Illumina sequencing, FastQC and FASTX-Toolkit have been used to detect abnormalities in a number of quality metrics. Next, reads were aligned to NCBI reference genome GRCh37 utilizing Tophat2^23^. The alignment quality was checked using SAMStat^24^, Picard-tools , RNA-SeQC^25^, SeqMonk, IGV^26^ and QualiMap^27^. The gene count was generated by HTSeq^28^, the results were used as input for DEseq2^29^ to analyze differential gene expression. Genes overexpressed in FF or FFPE tumor samples, relative to FF normal fallopian tube samples, were used for pathway analysis by the DAVID bioinformatics tool^30^. R and SeqMonk were used to calculate the gene expression correlation. Finally, a modified version of the pipeline SNPiR^31^ was used to analyze sequence variations in RNA sequencing data sets. Plots in Fig. 1 are generated using QualiMap, and plots in Figs 2, 3, 4, 5, 6 are generated with R scripts.

#### SNV calling from whole exome sequencing (WXS)

We used bwa (v0.6.2) aligned with seed length of 25 and default parameters, piping into samtools (v0.1.8) to sort. Additional sorting and adding read groups were processed with Picard-tools (v1.77). All bam files were then merged with samtools. The resulting merged file was processed with Picard-tools for PCR duplicate removal and with GATK (v2.4-7) for realignment, base recalibration, reducing of reads for faster variant calling. The comparison of SNV calls from DNA sequencing of corresponding FF and FFPE samples are reported elsewhere (Munchel and Hoang *et al.*, 2015)^32^.

GATKs UnifiedGenotyper was used to call SNVs.

## Results and Discussion

### Potential Artifacts in FFPE RNA Sequencing Data

In this study, we developed an analysis pipeline that incorporates various open-source tools, such as TopHat, RSEM, SNPiR, ANNOVAR, and custom filters to produce gene expression and single nucleotide variant calls from FFPE RNA sequencing data that are highly correlated and concordant with RNA sequencing data from patient-matched fresh frozen tumor samples (Figure S1). Quality control analyses of RNA sequencing data from FFPE tumor samples indicate results that are comparable with results from patient-matched FF tumor samples (Figure S2), with the exception of GC-content analysis. An abnormal peak around 53% GC content is conspicuous in all six FFPE samples (Fig. 1A), but not in FF samples (Fig. 1B). Visualization of reads from FFPE samples indicates that a large fraction of reads mapped to the intronic regions (Fig. 1C). Such an artifact is not observed in FF samples. These results suggest that reads mapping to the intronic regions may have contributed to the abnormal GC-content peak in FFPE samples. To confirm that, we applied a filter that removes reads outside of mRNA regions (mRNA regions downloaded from UCSC Table Browser), and the abnormal GC peak is no longer conspicuous in FFPE samples (Fig. 1D). This filter has no effect on FF samples (Fig. 1E). Although the total number of reads obtained from FFPE RNA libraries are higher, the total number of reads mapping to exons are lower in FFPE RNA libraries compared to FF RNA libraries (Fig. 2).

Capturing of intronic regions specifically in FFPE samples is most likely caused by the differences in sequencing library preparation between FF and FFPE RNA samples. Oligo-dT bound to magnetic beads was used to enrich mRNA species for FF samples, whereas total RNA and Ribo-Zero kit was used to deplete ribosomal RNA from FFPE RNA-sequencing libraries. Since RNA from FFPE samples are highly fragmented (Table S1) and thus not suitable for the enrichment of poly-A mRNAs with oligo-dT, total RNA library preparation followed by ribosomal depletion represents a suitable approach for FFPE RNA. Although it would be ideal to compare the results of RNA sequencing that utilizes the same ribosomal-depleted RNA library preparation method for both FF and FFPE samples, the objective of current study is to compare the results from most commonly used library preparation for FF samples with that for FFPE samples.

### Transcript Coverage

Coverage-over-transcripts plots are used to identify problems that may have occurred during the RNA library preparation or sequencing process. For example, a 3′-end bias in transcript coverage may indicate the degradation of RNA, because the reverse transcriptase would not be able to extend the reaction to the degraded 5′ end. A 5′-bias does not necessarily indicate a problem, but it may be viewed as a drop in 3′-coverage due to alternative poly-adenylation sites. Alternative poly-adenylation sites would result in missing coverage on the longer 3′-end of the reference transcripts.

The coverage plot over normalized transcript length for all six FF/FFPE pairs indicates a smaller, but discernible increase in 3′ coverage of all transcripts (Fig. 3A). To test if longer transcripts are more susceptible to 3′ coverage bias, transcripts were divided into the following three groups (Fig. 3B–D):

- short transcripts length (0.5–4 kbp); 27619 transcripts met this criterion (Fig. 3B)
- medium transcripts length (4–8 kbp); 8530 transcripts met this criterion (Fig. 3C)
- long transcripts length (>8 kbp); 1521 transcripts met this criterion (Fig. 3D)

Results in Fig. 3A indicate that coverage along the normalized transcript length is more consistent in FFPE samples than in FF samples. All FFPE samples have uniform coverage distributions, independent of the transcript length. In addition, we did not observe any influence of the expression levels on coverage bias along the transcript length in FFPE samples (Figure S4). In contrast to FFPE samples, with increasing transcript length, we observe a more pronounced 3′ bias in FF samples, with the lone exception of samples FF2938 (Fig. 3B–D), which also has the highest RIN number among the FF samples (Table S1). In addition, FF2474 and FF3356 have pronounced 3′ bias for medium and long transcripts, and these samples have the lowest RIN numbers (Table S1). These results collectively suggest that high quality RNA is needed to minimize 3′ bias in RNA library preparations that utilize oligo-dT capture. These results suggest that random priming reverse transcription of RNA from FFPE tumor samples effectively eliminates the 3′ bias expected from the heavily degraded RNA. In addition, the results also suggest that longer transcripts from FF samples are susceptible to degradation, and these transcripts may have been degraded at the 5′ during or prior to oligo-dT capture, thereby contributing to a drop in 5′ coverage.

### Gene Expression Correlation

We then used the NanoString technology as an orthogonal verification platform to determine the extent of correlation in gene expression between FF and FFPE samples (Figure S5). The gene count from both technologies and storage types has been quantile normalized, before calculating the correlation. Figure 3 visualizes the correlation matrix as a clustered heat map. Clusters are represented as a dendrogram. The color code has been adjusted according to the highest and lowest correlation value. Correlation boundaries, where both matching samples come from the same technique (RNA-sequencing or NanoString), are highlighted with a white frame. Correlation boundaries, where both matching samples come from different techniques (RNA-sequencing and NanoString), are highlighted with a blue frame.

All matching FF and FFPE samples cluster together (Fig. 4). These results indicate that the correlation between matched FF and FFPE samples are stronger than the correlation between unmatched samples. The correlation of gene expression between matched FF and FFPE samples quantified with NanoString technology is also very high. Even matched samples from RNA sequencing vs the NanoString (blue frame) show a distinctly better correlation than non-matching samples. Because the correlation between the NanoString and RNA-sequencing is higher in sample 3356 than for any other pair, both techniques cluster together instead of clustering with each particular technique. These results show that matched FF and FFPE samples share a strongly correlated gene expression, suggesting that FFPE samples can be a suitable replacement for FF samples in gene expression studies.

### Differential Gene Expression

The differential gene expression of six pairs of FF and FFPE samples was calculated utilizing DEseq2. RNA-sequencing data from seven normal fallopian tube samples was used as reference. The results show a very strong correlation of fold-change, relative to normal samples, in gene expression between matched FF and FFPE tumor samples (Fig. 5A). To determine the extent to which gene expression levels affect the correlation of gene expression between matched FF and FFPE pairs, we generated a MA-plot, where we plotted average gene expression on the x-axis and gene expression difference on the y-axis (Fig. 5B). We observed a marginally higher gene expression in FF samples for transcripts with log2-expression values less than 10 (Fig. 5B inset). These results suggest that less abundant transcripts are most likely to be affected by FFPE processing than the longer and more abundant transcripts. It is important to note that, although longer transcripts are more susceptible to degradation, the remaining degraded fragments can still be reverse-transcribed by random primers. In contrast, shorter and less abundant transcripts may be degraded to a state in which random priming is not possible. Another factor that may contribute to the observed bias in gene expression of low abundant transcripts is the reduced fraction of mapped reads to coding regions for the FFPE data sets.

We then performed differential gene expression analysis between FFPE tumor samples and FF normal samples as well as between FF tumor and FF normal samples. P-values of each transcripts were compared between FF and FFPE sample groups in relationship to the average level of gene expression (Fig. 5C). We do not observe any effect of gene expression levels on the differences of p-value. There is only a marginal p-value difference for each transcript between FF and FFPE samples, indicated by the regression smoothing line.

Four hundred seventy out of 742 (63.3%) genes, upregulated in FF tumor samples with reference to normal samples, were also upregulated in FFPE samples. One hundred fifty one out of 270 (55.9%) genes downregulated in FF tumor samples were also downregulated in FFPE samples. To perform pathway analysis, two lists of up-regulated genes from FF and FFPE samples were used as inputs for DAVID (Database for Annotation, Visualization and Integrated Discovery) analysis^30^. Approximately 50% of pathways identified in each storage group were also identified in the other storage group (Table S2).

### Mutation Analysis

Finally, a modified version of the pipeline “SNPiR” was used to analyze base changes in FF and FFPE RNA sequencing data sets. Mutations in RNA transcripts were called from both storage types (FF and FFPE), and the results were compared to mutations found in corresponding FF DNA sample by whole exome sequencing analysis. To visualize the comparison, for each sample the RNA variant allele frequency (variant detection rate) was plotted on the left y-axis and the DNA variant allele frequency (mutation rate) was plotted on the right y-axis (Fig. 6A). Each line represents a variant called in the RNA sample. The lines have color codes depending on the coverage and (in-)consistency of reported sequence variation. Variants with at least 10% allele frequency found in both RNA and DNA samples are colored green and called concordant if the DNA coverage is at least 10X for the base position. If the DNA coverage is below 10X for a certain base position, the variant is classified as ambiguous and indicated as a dotted line. Lines are colored red for discordant calls if the DNA coverage for the base position is at least 10X and the mutations are found only in RNA (AF>10%) and not in DNA (AF ≤ 10%).

Initially, FFPE samples produced a large number of discordant/false positive mutation calls (Fig. 6B). The majority of these false positive calls are most likely generated by formalin fixation process because the characteristic FFPE-prone C>T or G>A substitutions were significantly higher in FFPE samples than in FF samples (Fig. 6C). Box plot analysis of concordant and discordant C>T or G>A substitutions indicates these FFPE artifacts are present in FFPE samples at variant frequencies less than 50% (see Figure S6). We were therefore able to effectively eliminate these artifacts by applying a hard filter of ≥50% variant allele frequencies only for C>T or G>A substitutions. It is important to note that filtering these artifacts removed most of the discordant variant calls with little effect on concordant calls (Fig. 6D and Table 1).

These results show that RNA sequencing from FFPE samples can be used to identify high-confident single-nucleotide variations in tumor samples. It is important to note that neither FF nor FFPE RNA sequencing will pick up all mutations that can be found in DNA due to missing coverage in RNA (e.g. not expressed gene) or as a result of limitation in detecting mutations around splice sites from RNA sequencing data. FFPE samples have a false positive rate of approximately 4%. The slightly higher false positive rate is most likely caused by the formalin fixation process.

## Conclusion

RNA sequencing analysis indicates that gene expression between matched FF and FFPE pairs are strongly correlated, indicating the feasibility of using FFPE samples as a suitable replacement for FF samples. These results are consistent with prior studies by Hedegaard *et al.*^22^, indicating a strong correlation in gene expression between paired FF and FFPE samples. Our study took further steps by comparing differential gene expression between paired FF and FFPE tumor samples relative to FF normal samples and sequence variations from RNA sequencing data. These analyses indicate that fold-change in gene expression relative to normal tissues and sequence variations are strongly correlated between matched FF and FFPE pairs.

The observed overlap of approximately 50% in differential gene expression and pathway analysis may seem less impressive. However, considering that differential gene expression analysis of two randomly assigned groups within FF samples showed similar percentage of overlap in differentially expressed genes relative to 7 normal samples (data not shown), it is important to recognize the heterogeneity between tumor samples and the limitation of small sample size in current study that may be contributing to lower overlap in differentially expressed genes between FF and FFPE samples. Finally, our analysis demonstrates that FFPE sequence variation artifacts can be effectively removed by applying an allele fraction filter to C>T and G>A substitutions. Information will be useful for investigators planning to include FFPE samples for translational and clinical genomic studies.

## Additional Information

**How to cite this article**: Graw, S. *et al.* Robust gene expression and mutation analyses of RNA-sequencing of formalin-fixed diagnostic tumor samples. *Sci. Rep.* **5**, 12335; doi: 10.1038/srep12335 (2015).

## Supplementary Material

Supplementary Information

## Figures and Tables

**Table 1:** Concordance of SNV calls from FF and FFPE RNA sequencing compared to exome sequencing of FF DNA.

**Sample ID**	**FF concordant**	**discordant**	**FFPE concordant before filter**	**after filter**	**discordant before filter**	**after filter**
2474	161	0	223	222	125	17
2561	126	0	146	145	208	6
2938	179	2	224	224	108	6
3356	225	3	224	222	33	5
4079	281	0	215	215	128	6
4191	297	0	169	169	82	19
total	1269	5	1201	1197 (99.67%)	684	59 (−91.14%)

This table shows the efficiency of the applied filter to remove characteristic FFPE artifacts (C>T mutation at a low rate). Both, concordant and discordant, FFPE mutations are shown before and after applying the filter.
