# Supplementary material for: Robust gene expression and mutation analyses of RNA-sequencing of formalin-fixed diagnostic tumor samples
Source: Sci Rep. 2015 Jul 23;5:12335. doi: 10.1038/srep12335 (PMC4511951; doi:10.1038/srep12335)
Supplement: Supplementary Information [file srep12335-s1.pdf]

Robust gene expression and mutation analyses of RNA-sequencing of formalin-fixed diagnostic tumor samples

Stefan Graw[1,6], Richard Meier[1,6], Kay Minn[1], Clark Bloomer[2], Andrew K Godwin[3], Brooke Fridley[4], Anda Vlad[5], Peter Beyerlein[6], and Jeremy Chien[1,*]

[1]Department of Cancer Biology, University of Kansas Medical Center, 3901 Rainbow Blvd, Kansas City, Kansas 66160, USA
[2]Department of Laboratory Medicine and Pathology, University of Kansas Medical Center, 3901 Rainbow Blvd, Kansas City, Kansas 66160, USA
[3]Genome Sequencing Facility, University of Kansas Medical Center, 3901 Rainbow Blvd, Kansas City, Kansas 66160, USA
[4]Department of Biostatistics, University of Kansas Medical Center, 3901 Rainbow Blvd, Kansas City, Kansas 66160, USA
[5]Department of Obstetrics, Gynecology & Reproductive Sciences, University of Pittsburgh, 4200 Fifth Ave, Pittsburgh, Pennsylvania 15260, USA and
[6]Department of Bioinformatics and Biosystems Technology, Technical University of Applied Sciences Wildau, Hochschulring 1, 15745 Wildau, Germany

ADDITIONAL/SUPPLEMENTARY FILES

Script for workflow can be downloaded at:
www.kumc.edu/school-of-medicine/cancer-biology/faculty/jeremy-chien-phd/agct/publications.html

Figure S1. Analysis Workflow. Panel (A): Standard workflow for differential gene expression analysis. Panel (B): A custom workflow to analyze sequence variations from RNA sequencing data from FFPE and FF tumor samples.

Figure S2. Quality Control. Panel A (FF) and Panel B (FFPE): Top Figure: Base distribution of each position in all reads; Middle Figure: Base quality of each position in all reads; Bottom Figure: Duplication level, indicating how many sequenced reads are unique (Duplication Level = 1), how many sequenced reads are duplicates (Duplication Level = 2), how many sequenced reads are present three times (Duplication Level = 3), etc.; also other quality controls have been performed with similar results (data not shown)

Figure S3. Coverage along normalized transcript length for transcripts with varying levels of gene expression. The effect of gene expression levels on coverage along normalized transcript length on gene expression. The coverage of the 1000 highest (panel A), lowest (panel C) and medium (panel B) expressed genes has been analyzed. The samples are color coded (e.g. FF2474&FFPE2474: blue), while the line type is depending on the storage type (FF: solid; FFPE: dashed).

Figure S4. Scatter plot of gene expression from matched FF and FFPE pairs. A. A scatter plot of gene expression quantified by RNA sequencing from a representative sample 2474. The gene expression values for FF samples is on the x-axis and the gene expression value for FFPE samples is on the y-axis. The correlation value (R) indicates the degree of correlation between the two samples. The color key (blue to red) indicates the density of genes expression values from RNA sequencing. **B**. A scatter plot of gene expression quantified by NanoString from two representative pairs. Spearman correlation of gene expression within technical replicates and between unrelated samples are shown as a heatmap.

Figure S5. Box plot of allele frequency of C>T/G>A mutations of FFPE. The box plot shows the allele frequency distribution of concordant (same as DNA sequencing result) and discordant (different from DNA sequencing result) variant calls for C>T/G>A substitutions found in FFPE samples. The results indicate that allele fraction <0.5 will filter out almost all the discordant calls in FFPE RNA samples.

Table S1. List of matching tumor samples that were used for sequencing, differential gene expression, and mutation analysis

Sample	RNA				FFPE	DNA	Age of FFPE (years)	
ID	FF	RIN number	FFPE	RIN number	DV_{200}	FF		Histology
2474	FF2474	3.8	FFPE2474	2.3	74%	FF2474	< 1.5	Undifferentiated Carcinoma
2561	FF2561	6.2	FFPE2561	2.2	62%	FF2561	< 1.5	Papillary serous carcinoma
2938	FF2938	8.0	FFPE2938	2.8	75%	FF2938	< 1.5	Papillary serous carcinoma
3356	FF3356	4.7	FFPE3356	2.3	68%	FF3356	< 1.5	Papillary serous tumor, borderline
4079	FF4079	7.4	FFPE4079	2.3	58%	FF4079	< 1.5	Papillary serous carcinoma
4191	FF4191	7.3	FFPE4191	2.3	52%	FF4191	< 1.5	Papillary serous tumor, borderline

DV_{200} scale: >70% (Good); 50-70% (Moderate); 30-50% (Poor)

DV_{200}: Description of DV200 can be found at the Illumina website:

http://www.illumina.com/documents/products/technotes/technote-truseq-rna-access.pdf

Table S2. Common and unique pathways found in FF and FFPE samples deriving from differential gene expression analysis (seven normal fallopian samples used as reference).

Only FF	Common	Only FFPE
Regulation of actin cytoskeleton	Insulin signaling pathway	TGF-beta signaling pathway
Long-term potentiation	Arachidonic acid metabolism	Biosynthesis of unsaturated fatty acids
Drug metabolism	Cell adhesion molecules (CAMs)	Glycosaminoglycan degradation
Melanoma	Chemokine signaling pathway	Amino sugar and nucleotide sugar metabolism
Phenylalanine metabolism	Vascular smooth muscle contraction	Melanogenesis
PPAR signaling pathway	Focal adhesion	Gap junction
Tyrosine metabolism	Glycerolipid metabolism	Intestinal immune network for IgA production
ABC transporters	Complement and coagulation cascades	Ether lipid metabolism
Amyotrophic lateral sclerosis (ALS)	VEGF signaling pathway	Bladder cancer
Dorso-ventral axis formation	Type II diabetes mellitus	Sphingolipid metabolism
Long-term depression	Tryptophan metabolism	Ribosome
Glioma	MAPK signaling pathway	Renin-angiotensin system
Androgen and estrogen metabolism	Neuroactive ligand-receptor interaction	Adipocytokine signaling pathway
ECM-receptor interaction	Neurotrophin signaling pathway	Notch signaling pathway
	Calcium signaling pathway	Aldosterone-regulated sodium reabsorption
	Arginine and proline metabolism	
	Cardiac muscle contraction	

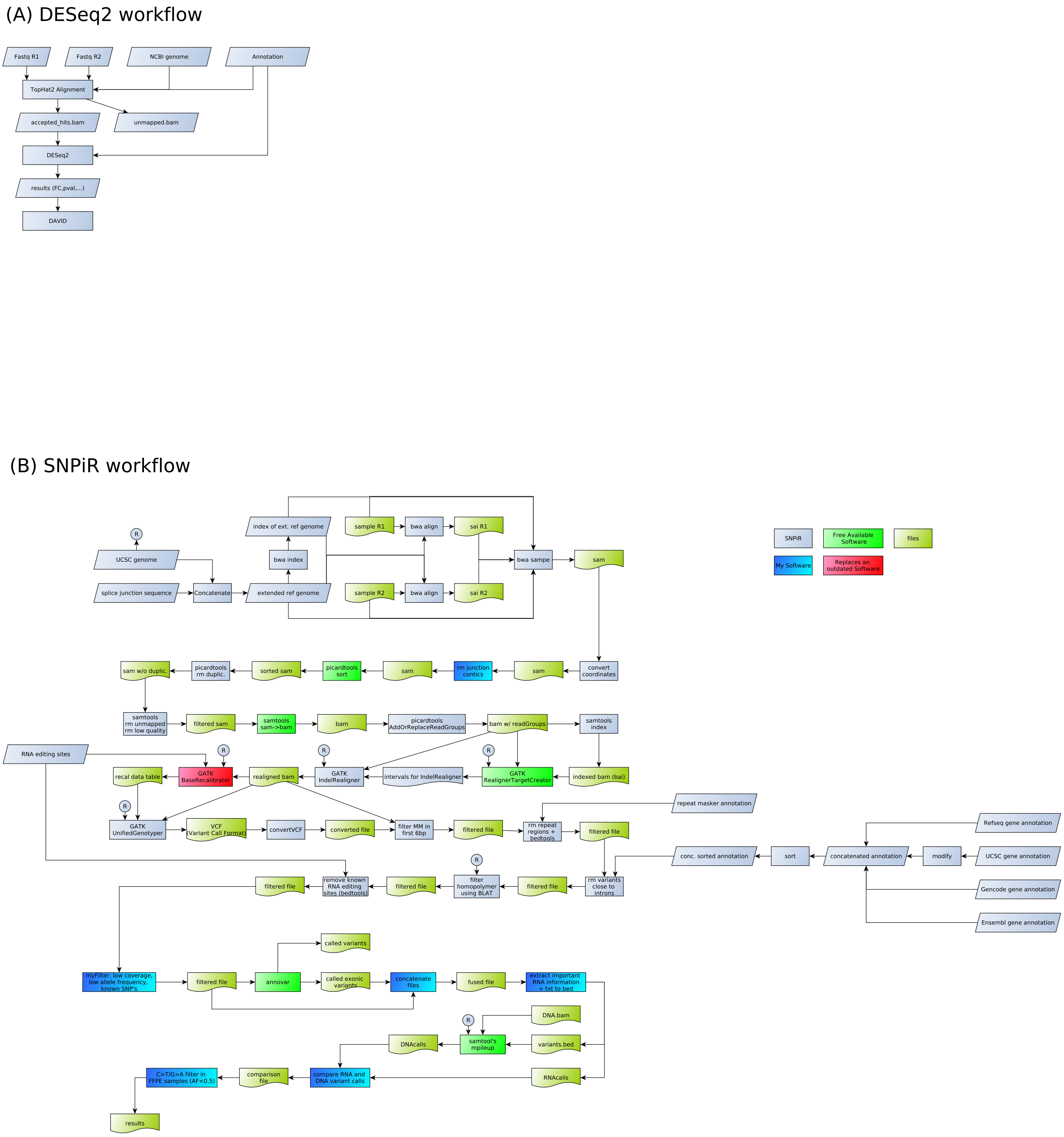

Figure S1: Analysis Workflow

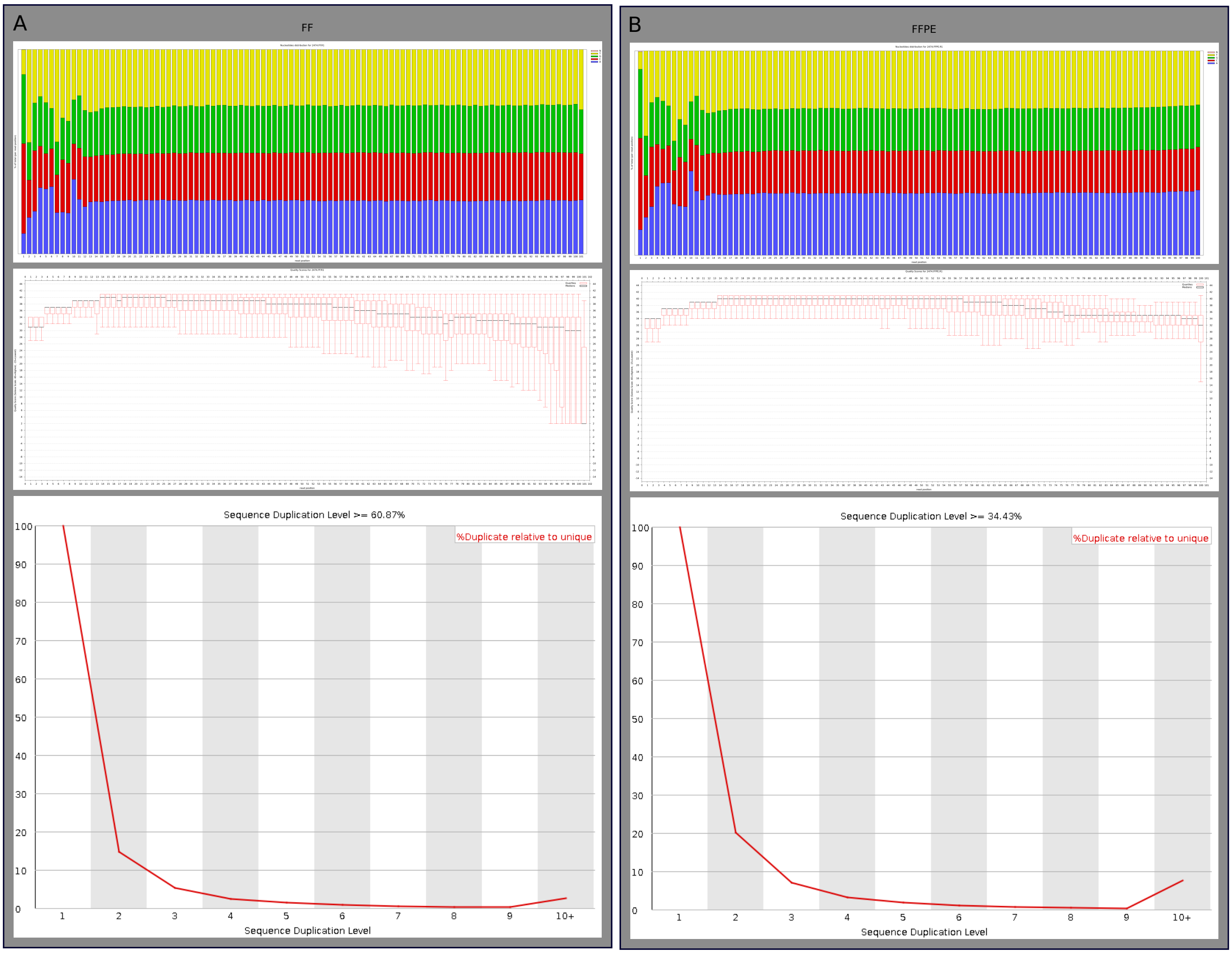

Figure S2: Quality Control

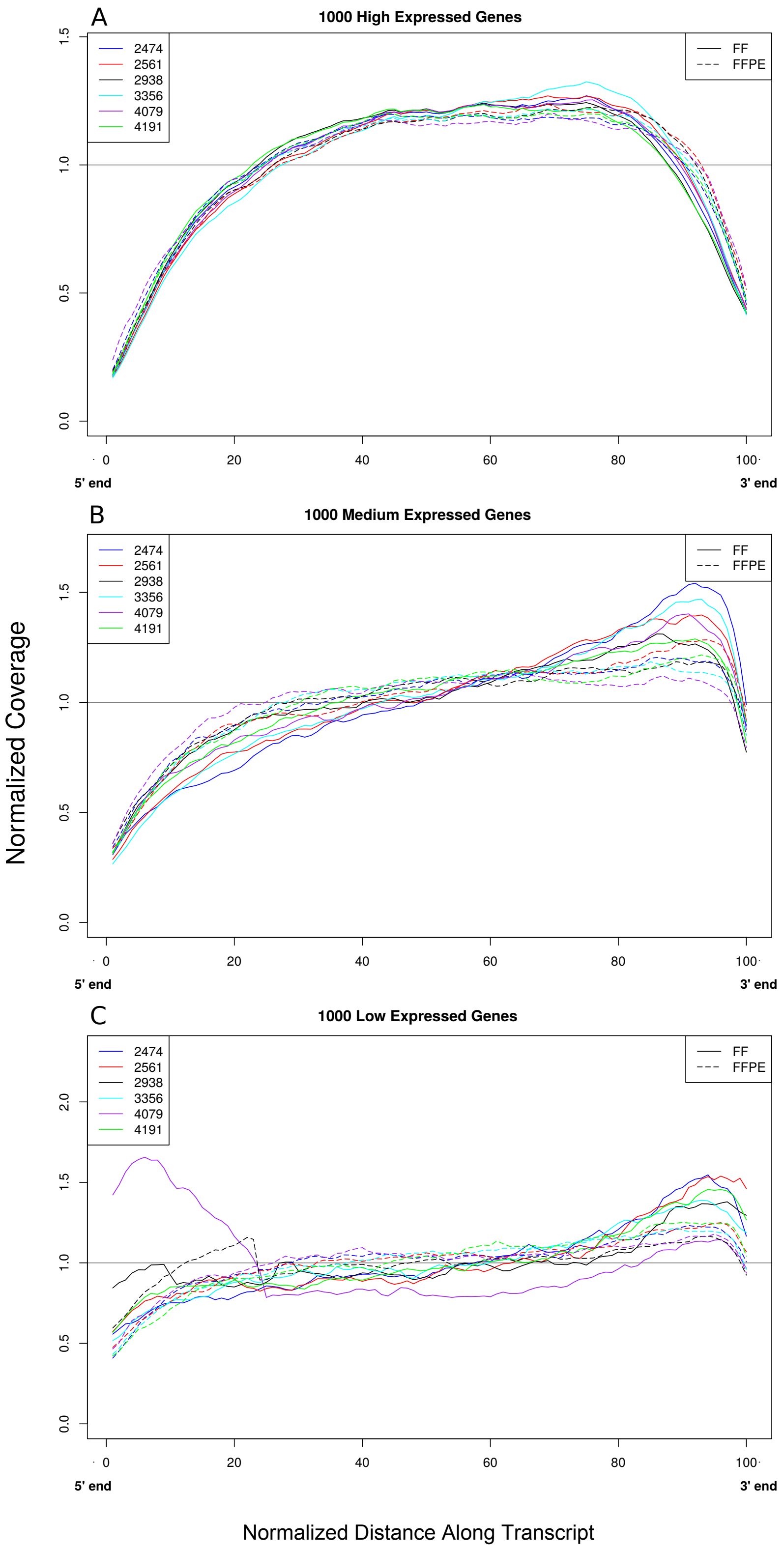

Figure S3: Coverage along normalized transcript length for transcripts with varying levels of gene expression

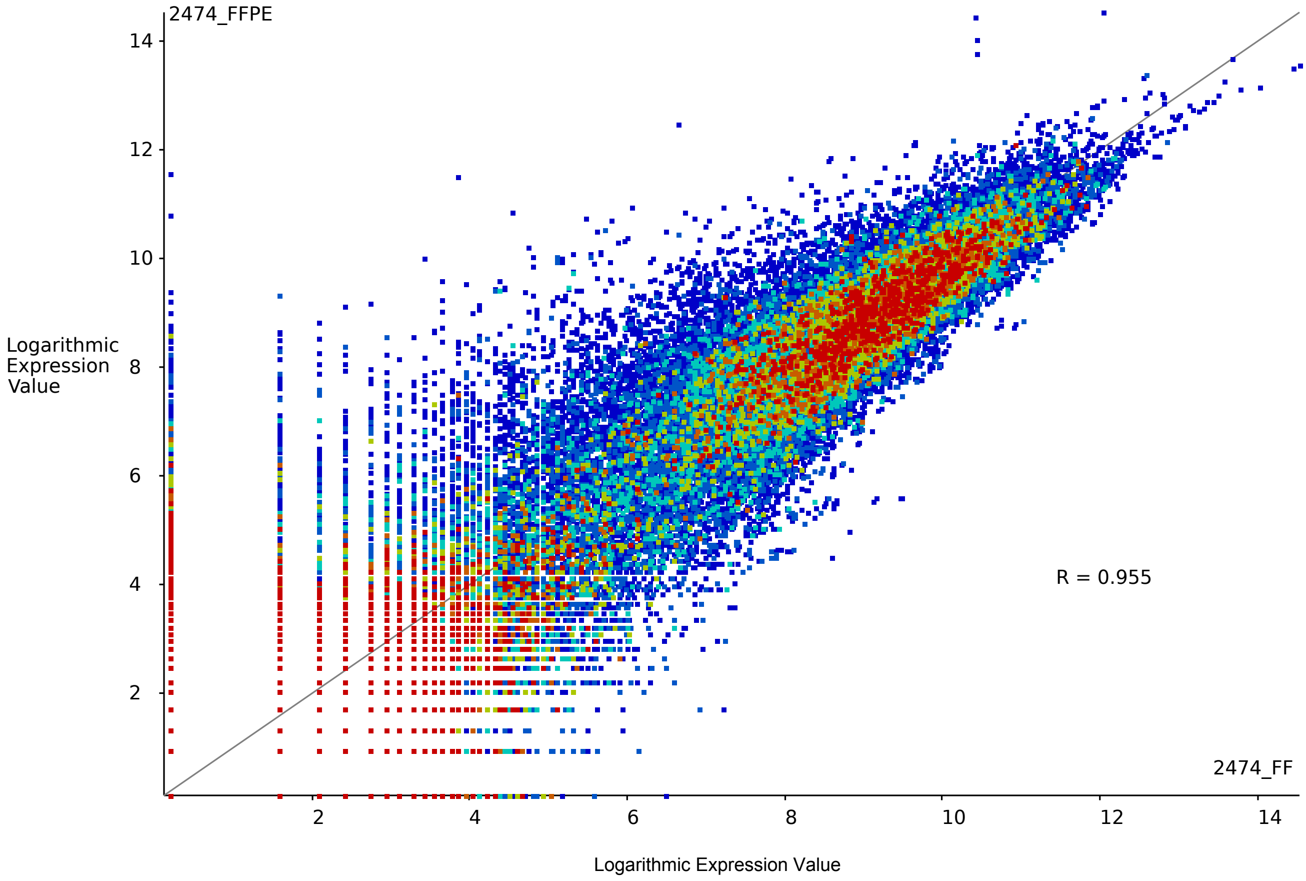

Figure S4A: Scatter plot of gene expression quantified from RNA sequencing data for matched FF2474 and FFPE2474 samples.

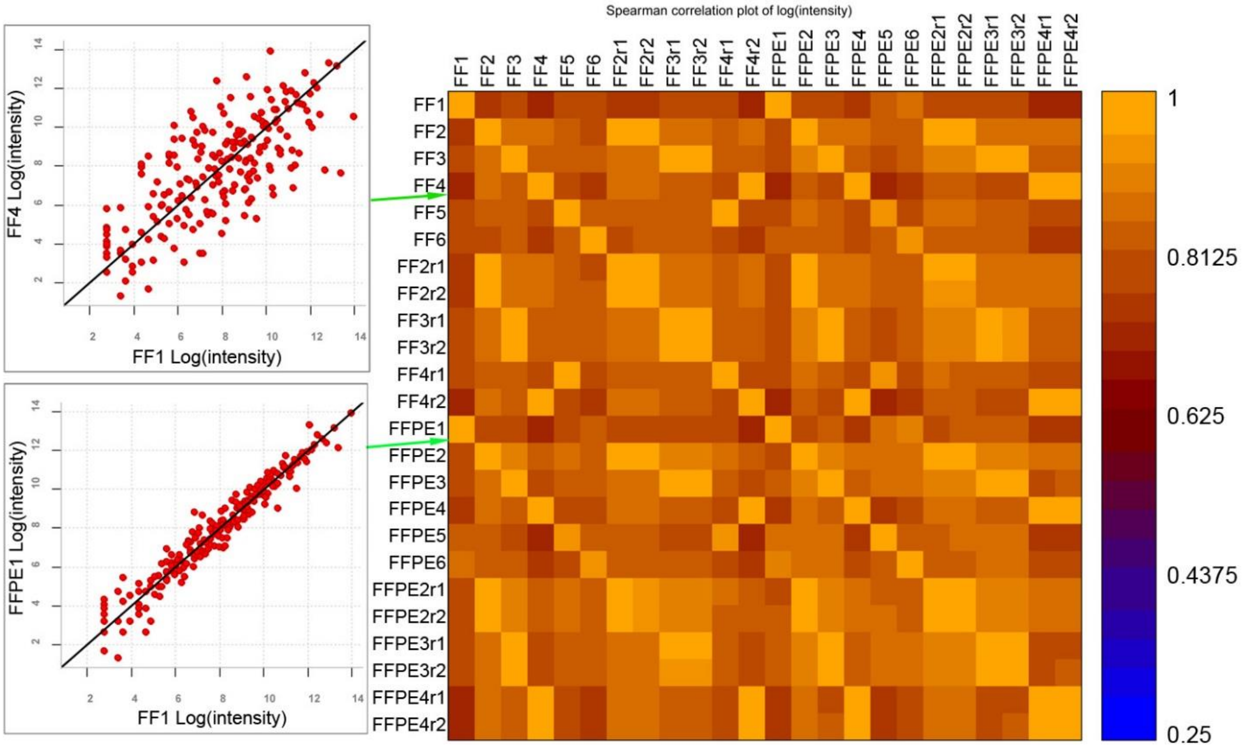

Figure S4B. Scatter plot of gene expression quantified by NanoString from matched FF and FFPE tumor samples. Spearman correlation within technical replicates and between different tumor specimens is shown as a heatmap.

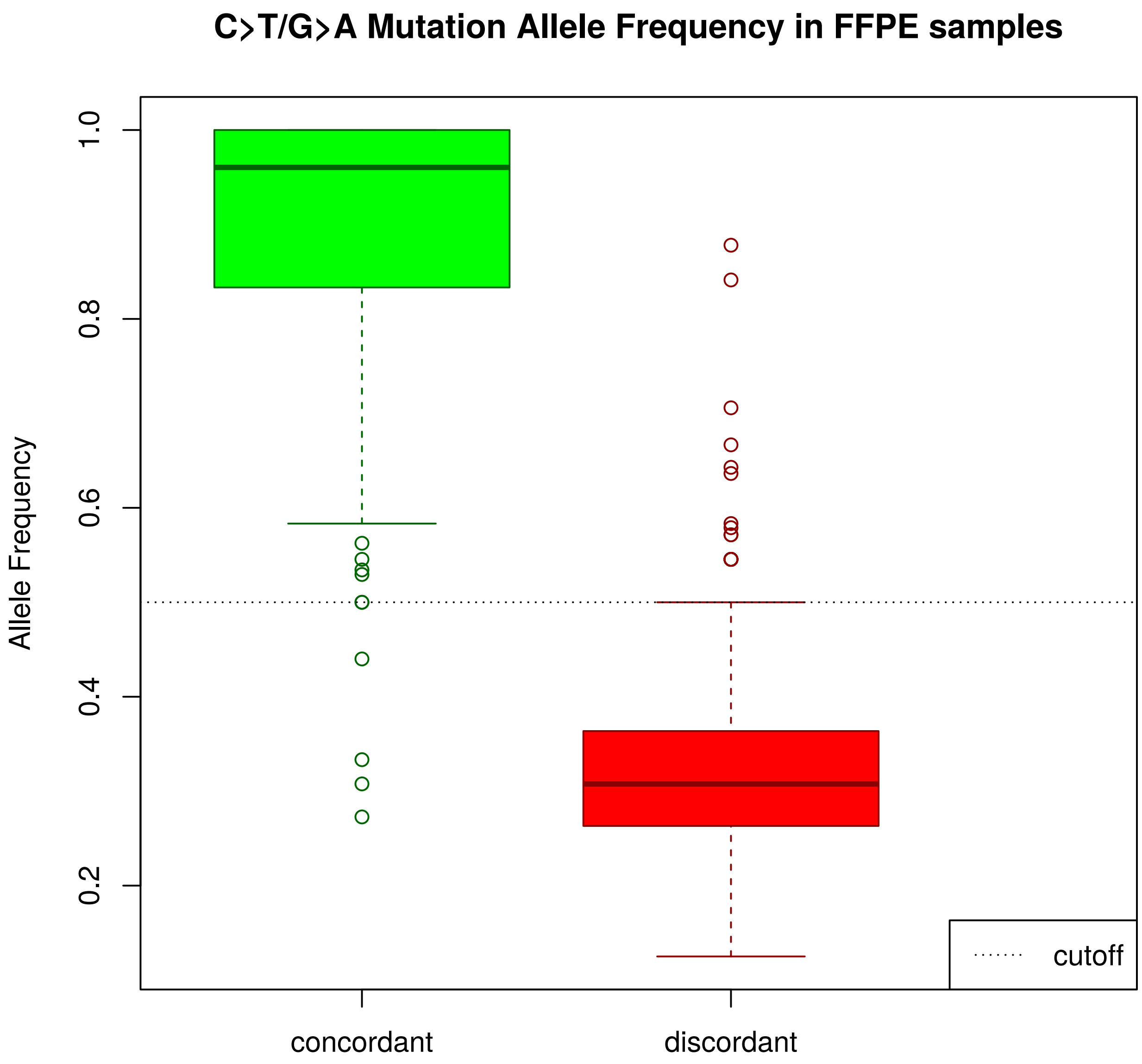

Figure S5: Box plot of allele frequency of C>T/G>A mutations of FFPE
